# Microbial processes during deposition and diagenesis of Banded Iron Formations

**DOI:** 10.1007/s12542-021-00598-z

**Published:** 2021-12-08

**Authors:** Carolin L. Dreher, Manuel Schad, Leslie J. Robbins, Kurt O. Konhauser, Andreas Kappler, Prachi Joshi

**Affiliations:** 1 Geomicrobiology, Center for Applied Geosciences, University of Tuebingen, Tuebingen, Germany; 2 Department of Earth and Atmospheric Sciences, University of Alberta, Edmonton, AB Canada; 3 Department of Geology, University of Regina, Regina, SK Canada

**Keywords:** Banded Iron Formations (BIF), iron redox processes, Microbial iron cycling, Nutrients

## Abstract

Banded Iron Formations (BIFs) are marine chemical sediments consisting of alternating iron (Fe)*-*rich and silica (Si)-rich bands which were deposited throughout much of the Precambrian era. BIFs represent important proxies for the geochemical composition of Precambrian seawater and provide evidence for early microbial life. Iron present in BIFs was likely precipitated in the form of Fe^3+^ (Fe(III)) minerals, such as ferrihydrite (Fe(OH)_3_), either through the metabolic activity of anoxygenic photoautotrophic Fe^2+^ (Fe(II))-oxidizing bacteria (photoferrotrophs), by microaerophilic bacteria, or by the oxidation of dissolved Fe(II) by O_2_ produced by early cyanobacteria. However, in addition to oxidized Fe-bearing minerals such as hematite (Fe^III^_2_O_3_), (partially) reduced minerals such as magnetite (Fe^II^Fe^III^_2_O_4_) and siderite (Fe^II^CO_3_) are found in BIFs as well. The presence of reduced Fe in BIFs has been suggested to reflect the reduction of primary Fe(III) minerals by dissimilatory Fe(III)-reducing bacteria, or by metamorphic (high pressure and temperature) reactions occurring in presence of buried organic matter. Here, we present the current understanding of the role of Fe-metabolizing bacteria in the deposition of BIFs, as well as competing hypotheses that favor an abiotic model for BIF deposition. We also discuss the potential abiotic and microbial reduction of Fe(III) in BIFs after deposition. Further, we review the availability of essential nutrients (e.g. P and Ni) and their implications on early Earth biogeochemistry. Overall, the combined results of various ancient seawater analogue experiments aimed at assessing microbial iron cycling pathways, coupled with the analysis of the BIF rock record, point towards a strong biotic influence during BIF genesis.

## Introduction

**Fig. 1:**
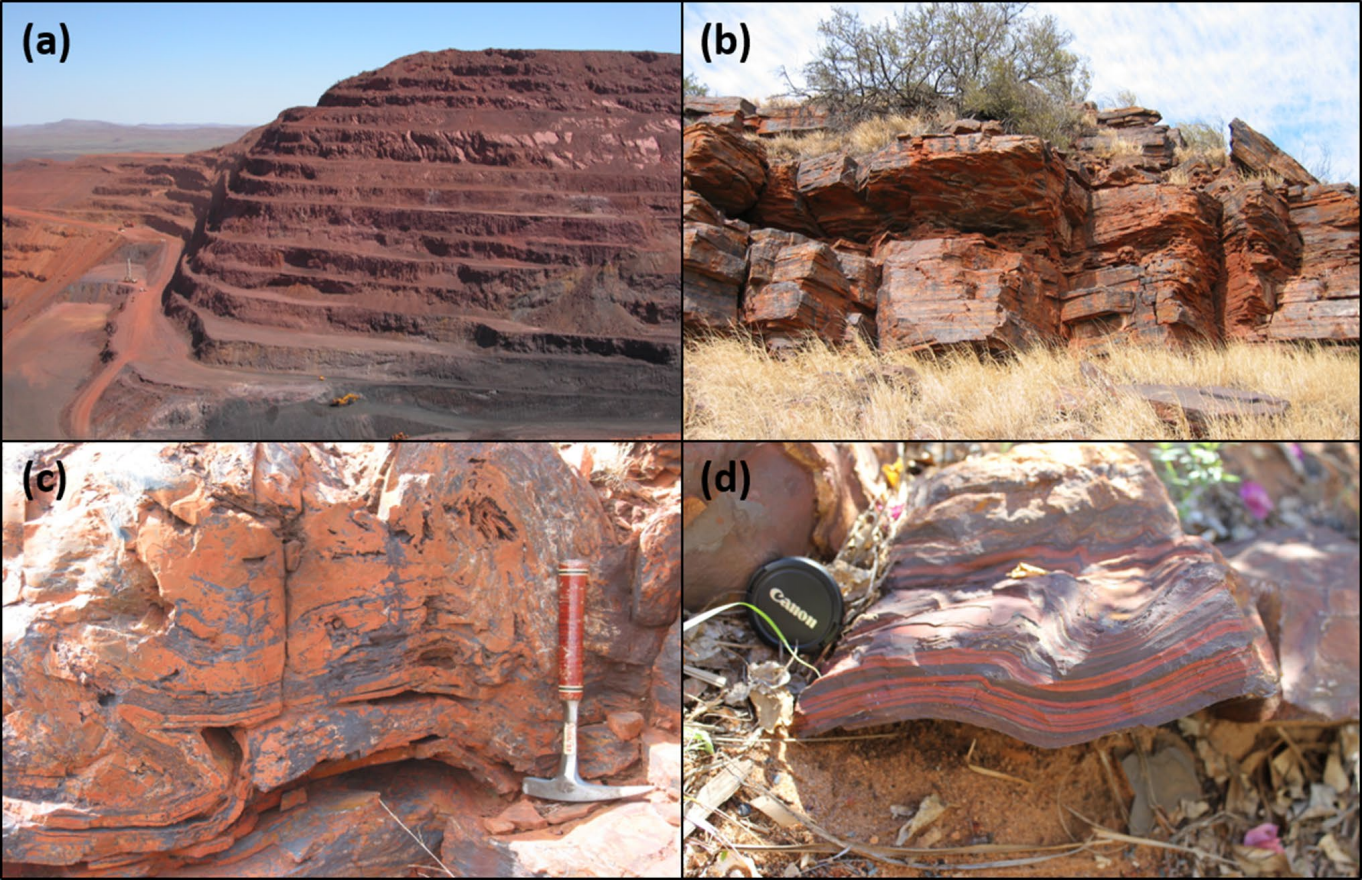
Photographs of Banded Iron Formations (BIFs); **a** view of a mine at the southern ridge of the Tom Price BIFs, Western Australia (provided by Mark Barley); **b** BIF formation in Gamohaan Hill near Kuruman, Northern Cape Province, South Africa; **(c)** close-up of (**b**), showing centimeter-thick alternating wavy bands of iron-rich and silica-rich bands; **(d)** alternating millimeter-thick bands of a Hamersley Group BIF sample, Western Australia (provided by Jan-Peter Duda)

Precambrian Banded Iron Formations (BIFs) (Fig. 1) are marine chemical sediments consisting of alternating iron oxide-rich and silica-rich bands (Trendall and Blockley 1970). BIFs can be found as massive iron ore deposits in Australia, South Africa, Canada, China, Brazil, Ukraine, and a number of other locations, with occurences noted on every continent (Bekker et al. 2014; Ernst and Bau 2021; Konhauser et al. 2017). For example, the Hamersley Group in Western Australia extends over ~ 10^5^ km^2^, and contains more than 10^13^ tons of iron (Beukes 1984; Trendall 2002). BIFs may be divided into two broad classes: Algoma-type BIFs that are associated with volcanic arc settings and Superior-type BIFs which were deposited on passive margins (see Bekker et al. 2014 and Konhauser et al. 2017 for reviews). Primarily, BIFs were formed between 3.8 and 1.85 Ga, with extensive Superior-type BIFs thought to have mainly been deposited on continental shelves and slopes at depths of less than 400 m (Trendall 2002), and smaller formations being deposited in the deep ocean (Krapež et al. 2003). Peak BIF deposition has been constrained to the Neoarchean and early Paleoproterozoic (Beukes 1984; Klein 2005; Konhauser et al. 2017; Trendall 2002), shortly preceding the Great Oxidaiton Event (GOE).

BIFs are of great interest as they reflect the (bio)geochemistry of the oceans in which they were deposited. In addition to acting as a proxy for paleomarine chemistry, BIFs also contain evidence of the first significant rise in atmospheric oxygen during the GOE between 2.45 and 2.32 Ga (Bekker et al. 2004; Buick 2008; Farquhar et al. 2011; Konhauser 2011; Pavlov and Kasting 2002; Rye and Holland 1998). Critical to using the BIF record to interpret early Earth conditions is an understanding of formation pathways: were BIFs formed abiotically or through the activity of microorganisms? Evidence of microbial influence on BIF deposition would indicate conditions favorable to life, specifically nutrient-rich oceans (Tyrrell 1999; Zahnle et al. 2006; Zerkle 2005) with circumneutral pH values (Cloud 1965, 1973). On the other hand, if BIF deposition and diagenesis were primarily abiotic processes, Precambrian oceans could have had more alkaline pH values (Beukes and Gutzmer 2008; Tosca et al. 2016) and may have been nutrient-poor (Bjerrum and Canfield 2002).

Here, we review potential pathways for BIF deposition with a particular focus on the role of microorganisms and the subsequent post-depositional alteration of iron minerals within BIFs via microbial activity. Then, we review examples of elemental cycling linked to the microbial Fe cycling during BIF deposition, specifically the macronutrient phosphorus and the trace element nickel. We close this review by suggesting potential future research directions that may aid in resolving ongoing controversies regarding the biological role in BIF deposition.

## Mineralogy of Banded Iron Formations (BIFs)

Banded Iron Formations (BIF) consist of alternating bands characterized by high iron oxide (20–40%) and high silica (40–60%) content (Bekker et al. 2014; Beukes 1984; Klein 2005; Konhauser et al. 2017; Trendall 2002). While the silica-rich layers are mostly composed of bedded chert (Rasmussen et al. 2017), the iron oxide-rich layers contain a wide range of iron-bearing minerals with an overall Fe oxidation state of 2.4 (Klein and Beukes 1992). The mineralogy observed in BIFs today is comprised of magnetite (Fe^II^Fe^III^_2_O_4_), hematite (Fe_2_O_3_), siderite (FeCO_3_) and other iron carbonates, Fe(II/ III)-silicates, and chert, among others (Bekker et al. 2014; Klein 2005). It is generally recognized that the current mineralogy is the result of diagenetic and metamorphic overprinting of the primary minerals deposited on the seafloor. Ferric oxyhydroxides (e.g. ferrihydrite, Fe(OH)_3_), iron silicates (e.g. greenalite, Fe_3_Si_2_O_5_(OH)_4_)), Fe(III)-silica gels, and iron carbonates (e.g., siderite, FeCO_3_) have each been hypothesized as the primary mineral phases that were precipitated in the water column and deposited on the seafloor depending on whether a biological or abiotic formation pathway is assumed (Alibert 2016; Dimroth and Chauvel 1973; Halevy et al. 2017; Han 1966, 1978; Johnson et al. 2018; Klein 2005; Percak-Dennett et al. 2011; Perry et al. 1973; Rasmussen et al., 2014, 2015a, b; Sun et al. 2015; Wu et al. 2012; Zegeye et al. 2012).

## Atmospheric and marine conditions during BIF deposition

The atmospheric and oceanic chemical conditions in the Precambrian were substantially different from the modern systems of today. Oxygen concentrations in the atmosphere and global oceans were negligible (Hardisty et al. 2014; Holland 2002; Olson et al. 2013; Pavlov and Kasting 2002). There was no ozone layer in the atmosphere prior to the GOE, resulting in UV radiation penetrating the top layers of the ocean (Bekker et al. 2004; Catling and Zahnle 2020; Konhauser et al. 2017; Lyons et al. 2014). Exceptions to the low oxygen concentrations in the oceans were coastal regions with oxygen produced by cyanobacteria, with assumed maximum oxygen concentrations varying between 5 µM and 100 µM (Czaja et al. 2012; Kasting 1992; Kendall et al. 2010; Planavsky et al. 2010). Geochemical models predict that Archean oxygen oases were characterized by oxygen concentrations on the order of 10 µM (Olson et al. 2013).

Geochemical processes in the marine environment were dominated by the complex interplay of the iron, silicon, and carbon cycles. The absence of silica-precipitating microorganisms allowed dissolved silica to accumulate to concentrations that approached saturation with respect to amorphous silica (0.67–2.2 mM) (Maliva et al. 2005; Siever 1992). Furthermore, the overall low oxygen concentrations in environments distal from cyanobacterial mats allowed the buildup of high concentrations of dissolved Fe(II) (0.02–0.5 mM) (Holland 1973; Morris 1993) sourced from hydrothermally derived fluids (Bau and Möller 1993; Hamade et al. 2003; Jacobsen and Pimentel-Klose 1988). This dissolved Fe(II) was potentially transported either by advection (current driven upwelling of deep waters onto continental shelves), with Fe(II) at depth supplied by mid ocean ridge systems (Holland 1973; Konhauser et al. 2007; Morris and Horwitz 1983), or by direct transport into the photic zone by hydrothermal plumes emanating from shallow seamount systems (Isley 1995; Isley and Abbott 1999).

## Deposition: primary iron mineral precipitation

The deposition pathways of BIFs are highly debated. For the precipitation of these alternating iron oxide-rich and silica-rich layers, high concentrations of dissolved Fe(II) in anoxic sea water had to be oxidized to dissolved Fe(III) and precipitated as Fe(III) minerals. The oxidation and subsequent precipitation processes have been suggested to occur purely abiotically by some studies, while other studies have argued that microorganisms are directly involved in the oxidation of Fe(II) and subsequent Fe(III) mineral precipitation. In principle, biotic and abiotic Fe minerals differ in chemical composition, particle size, and density (Posth et al. 2014), surface properties (Moon and Peacock 2012), and adsorptive behavior towards nutrients and trace metals (Sundman et al. 2016; Yan et al. 2016). These characteristics may be used to differentiate between the origin of freshly synthesized Fe minerals; however, since there are no primary Fe(III) minerals preserved that may be examined directly, it is difficult to reconstruct the origin and properties of the primary minerals precipitated from the water column. We discuss below the different chemical and biological hypotheses for BIF mineral formation based on evidence from field observations and laboratory experiments conducted under conditions representative of the early Earth.

## Abiotic deposition mechanisms

Based on the iron silicates present in BIFs today, some studies have argued that the origin of the primary minerals in BIFs was purely abiotic. Harder (1976, 1978) observed that nontronite or chamosite, two iron silicates observed in iron-rich formations, could be abiotically formed during anoxic, low-temperature synthesis of poorly crystalline Fe(III) oxyhydroxides from dissolved Fe(II) in the presence of silica. Harder (1976, 1978) and Konhauser et al. (2007) supported the formation of Fe-silicates in anoxic, non-sulfidic media with high dissolved Fe(II) and silica concentrations. Amongst the abiotic pathways, one of the proposed hypotheses suggests that Fe(II/III) silicates, particularly greenalite ((Fe^II^, Fe^III^)_2-3_Si_2_O_5_OH_4_), was the main primary iron mineral and that it was deposited as nanometer-sized mud particles or loose flocs due to co-nucleation of Fe(II) and silica (Rasmussen et al. 2013, 2015b, 2019a). According to this hypothesis, current BIF mineralogy would, therefore, be explained by diagenetic, metamorphic, or weathering overprinting (Rasmussen et al. 2019b). One limitation of this pathway is the high pH value (7.5–8) necessary for the formation of Fe silicates (Beukes and Gutzmer 2008; Tosca et al. 2016), which is at odds with the assumed slightly acidic to circumneutral pH of the ocean at that time (Halevy et al. 2017; Krissansen-Totton et al. 2018). Further, it has been shown that secondary oxidation of Fe-silicates, either through percolating oxidizing fluids or weathering, is not able to oxidize sufficient amounts of Fe(II) necessary to explain all the Fe(III) in BIFs on reasonable time scales (Robbins et al. 2019).

**Fig. 2:**
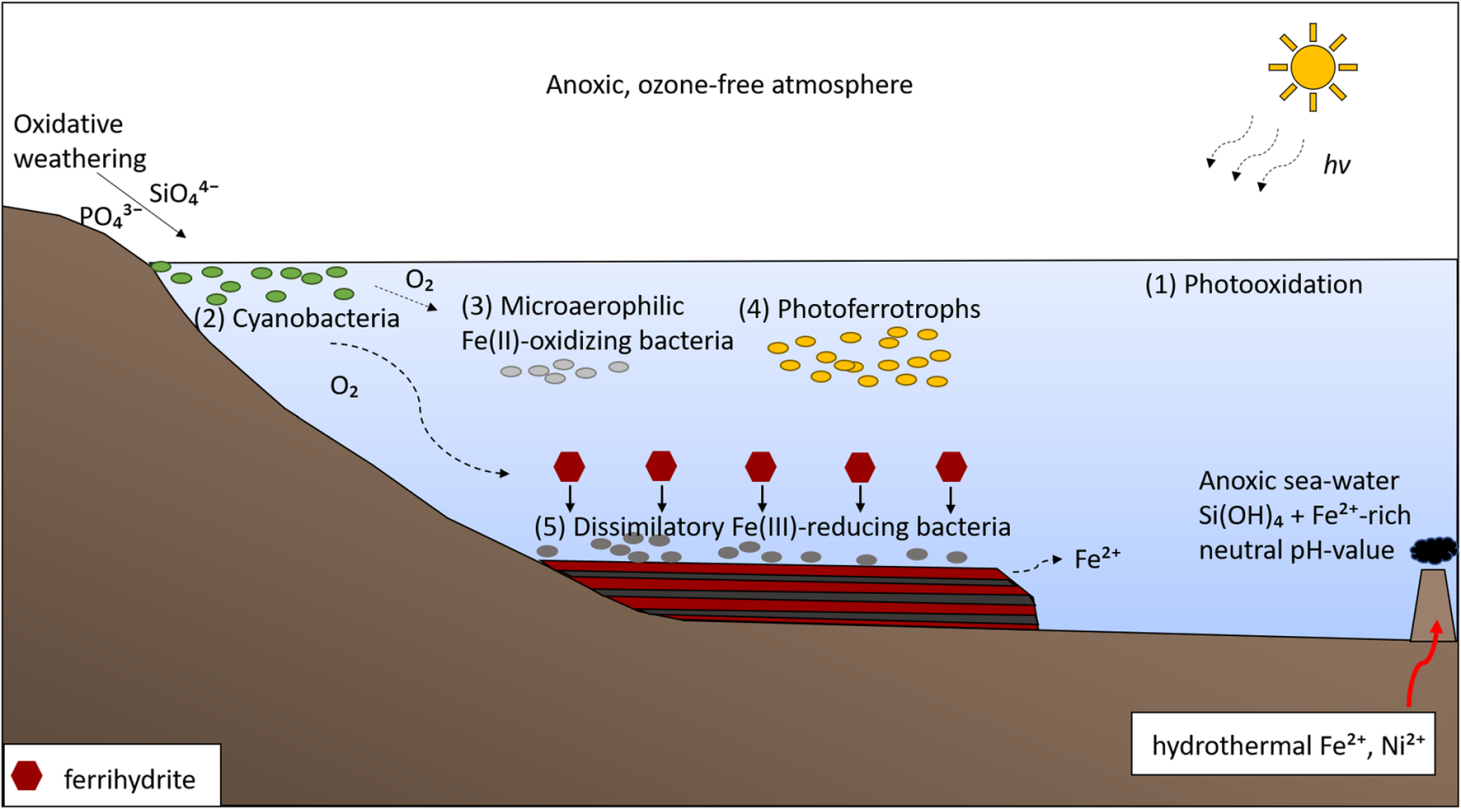
Overview of biotic and abiotic Banded Iron Formations (BIFs) deposition mechanisms. Precambrian seawater was anoxic, silica- and iron-rich. Silica and phosphate were transported following the weathering of rock formations, and Fe(II) and nickel were supplied by hydrothermal fluxes. **1.** Abiotic photooxidation of Fe(II) by UV radiation, directly leading to abiotic formation of Fe(III); **2.** Oxygen produced by cyanobacteria leading to abiotic oxidation of dissolved Fe(II) to form Fe(III) minerals; **3.** Microaerophilic Fe(II)-oxidizing bacteria reducing oxygen produced by cyanobacteria for direct oxidation of Fe(II)_(aq)_ to Fe(III) minerals; **4.** Direct oxidation of dissolved Fe(II) by phototrophic Fe(II)-oxidizing (photoferrotrophic) bacteria and **5.** partial reduction of deposited Fe(III) minerals to mixed-valence state Fe(II)Fe(III) minerals by dissimilatory iron(III)-reducing bacteria (DIRB)

A second prevalent hypothesis concerning the abiotic formation of BIFs is the photochemical oxidation of dissolved Fe(II) (Fig. 2:1). Due to the lack of an ozone layer in the atmosphere, solar UV radiation was not attenuated. Therefore, dissolved Fe(II) in the photic zone might have adsorbed UV light, at wavelengths from 200 to 400 nm, resulting in the photochemical oxidation of ~ 0.07 atoms of dissolved Fe(II) per photon (Nie et al. 2017) and the formation of Fe(III)

1. (Braterman et al. 1983; Cairns-Smith 1978)

1 $$ 2{\text{Fe}}^{2 + } \left( {{\text{aq}}} \right) + 2 {\text{H}}^{ + } + hv \to 2 {\text{Fe}}^{3 + } \left( {{\text{aq}}} \right) + 2{\text{H}}_{2} \uparrow $$

However, the photooxidation pathway has been challenged by experimental studies examining the effect of aqueous chemistry on UV photochemical oxidation. (Konhauser et al. (2007) observed experimentally that at high dissolved silica and bicarbonate concentrations, the photooxidation of Fe(II) by UV light was substantially inhibited. This inhibition would suggest that photochemical oxidation, compared to other deposition pathways, only played a minor role in BIF deposition.

## Biotic deposition mechanisms

The majority of past work looking at biological pathways of BIF formation has focused on the role of cyanobacteria (Fig. 2:2). Fe(III) has been hypothesized to be formed via the chemical oxidation of dissolved Fe(II) by oxygen produced by cyanobacteria under circumneutral pH conditions (Cloud 1965, 1973; Konhauser et al. 2007) and minerals such as ferrihydrite (Fe(OH)_3_) are presumed to have been the dominant primary mineral. Once free oxygen became available in the ocean, other microorganisms, such as neutrophilic microaerophilic Fe(II)-oxidizers (Fig. 2:3), may have evolved and further contributed to Fe(III) mineral deposition by direct (enzymatically driven) Fe(II) oxidation (Chan et al. 2016; Holm 1989).

There are several lines of evidence suggesting that BIF formation may not be limited to cyanobacteria (Garrels et al. 1973; Hartman 1984; Widdel et al. 1993). The assumption that cyanobacteria played a role in BIF deposition is partially founded on microfossils in the Precambrian sedimentary record. However, the dating of the first cyanobacterial occurrence in these formations is highly debated, as some of the oldest microfossils are not unambiguously primary or secondary in nature (Schirrmeister et al. 2016; van Kranendonk 2006). Additionally, cyanobacteria might have suffered from ultraviolet irradiation (Mloszewska et al. 2018)) and/or Fe(II) toxicity (Swanner et al. 2015a, c), and potentially severe nutrient (e.g., phosphorous) limitations (Jones et al. 2015). These factors would have presented significant obstacles for early cyanobacteria and raise questions regarding the role cyanobacteria had in early BIF formation.

Alternatively, Garrels et al. (1973), Hartman (1984) and Widdel et al. (1993) proposed that a different microbial process may have been responsible for the oxidation of the Fe(II), even under anoxic conditions: anoxygenic, photoautotrophic Fe(II) oxidation (photoferrotrophy) (Fig. 2:4). Photoferrotrophic bacteria use Fe(II) as an electron donor coupled to CO_2_ fixation in the presence of light, resulting in the precipitation of Fe(III) and the formation of biomass (Bryce et al. 2018). Their habitat reaches water depths of up to 100 m, facilitating BIF deposition even in the simultaneous presence of cyanobacteria in the overlaying water column (Kappler et al. 2005). Indeed, work by Konhauser et al. (2002) suggests that bacteria, and especially photoferrotrophic bacteria, could have deposited most, possibly even all, Fe contained in BIFs today. Although anoxygenic phototrophs may also be negatively affected by nutrient (e.g., phosphorus) limitations, this effect would be less severe than that on cyanobacteria (Jones et al. 2015). Another potential microbial metabolism contributing to direct Fe(III) precipitation, is nitrate-dependent Fe(II) oxidation (Weber et al. 2006). However, the presence of oxidized species, such as nitrate, in seawater is dependent on higher oxygen availability than assumed for Precambrian oceans (Oshiki et al. 2013). A major contribution to BIF deposition by these bacteria is, therefore, considered unlikely. Although indisputable conclusive evidence for the role of microorganisms in BIF deposition has not been found, there are several lines of direct and indirect evidence that support this hypothesis as discussed below.

## Evidence for early cyanobacteria and photoferrotrophs

The hypothesis of microbial influence in BIF deposition is based on the widespread theory that the GOE at 2.45 Ga was initiated by the mass occurrence of cyanobacteria (Bekker et al. 2004; Holland 2002). Critical to this hypothesis is the presence of microbes during the time of BIF deposition, which has been hypothesized mainly based on evidence of microorganisms (in this case, cyanobacteria and photoferrotrophs) in contemporaneous formations. The first robust evidence for the presence of widespread microbial life dates back to at least 3.5 Ga and includes stromatolites (Baumgartner et al. 2019; Duda et al. 2016; Mißbach et al. 2021; van Kranendonk 2006; Wacey et al. 2006) and geochemical information such as the stable carbon compositions and molecular evidence in sedimentological kerogens (Duda et al. 2018; Hayes 1965; Schidlowski 2001). Although the metabolisms are not always clear, some of these early mats show several lines of independent evidence that point towards the existence of phototrophic bacteria, like phototactic growth (growth towards the sun), highly negative δ^13^C values and molecular clocks (a method to date the evolution of life by comparing DNA mutations of biological groups) (Allwood et al. 2009; Schirrmeister et al. 2016). Hence, cyanobacteria are a possible source of oxygen and, consequently could be (partly) responsible for the formation of BIFs.

So far, no microfossil evidence for photoferrotrophic bacteria in the Archean ocean had been documented (Posth et al. 2013). Chi Fru et al. (2013), reported fossil evidence of an ancient relative of the photoferrotroph *Rhodomicrobium Vannielii* in a quaternary BIF analogue, a hematite-rich jasper bands from Cape Vani in the North West of Milos, Greece. Besides microfossils, research has focused on phylogenetics and ecophysiology, to examine the evolution of photoferrotrophs and the ability to adapt to different living conditions. Woese (1987) suggested that all known phototrophic strains descended from one common ancestor, which likely evolved before oxygenic cyanobacteria (Xiong 2006). Based on experimental findings and the monitoring of recent representative strains, it has been inferred that photoferrotrophs are able to adapt to a wide variety of iron-rich environments (Croal et al. 2009), suggesting that they would have been able to survive in the ancient ocean.

## Stable isotope ratios as evidence for early microbial life

Although the state of evidence for microbial life based on biomarkers and especially the microbial role in BIF formation is not unambiguously established, stable isotope ratios in the ancient rock record can support arguments for microbial life. Chemical and biological processes often influence the stable isotope composition of the geochemical phases involved; as a result, relative enrichment or depletion of specific isotopes in the product relative to the initial reservoir allows us to infer the underlying reactions. In the case of microbial metabolism, autotrophic (CO_2_-fixing) activity is usually marked by a depletion of ^13^C (relative to ^12^C) in the metabolic product (e.g., organic matter). To compare the depletion of different proxies, the ratio of ^13^C to ^12^C compared to a standard is used, termed “δ^13^C”. Therefore, highly depleted organic δ^13^C values (from ~  − 10 up to − 35 ‰) may indicate microbial autotrophic processes, especially photosynthesis (Schidlowski 2001). Several 3.5 to 3.2 Ga examples of Archean kerogens display such ^13^C depletions: some formations in Western Australia and South Africa show isotopic values from − 20 to − 35 ‰ (Altermann and Kazmierczak 2003; Tice and Lowe 2004) and samples from the late Archean, 2.59 to 2.5 Ga old Transvaal Supergroup have organic δ^13^C ranging from − 25 to − 40 ‰ (Fischer et al. 2009). Further evidence of early microbial life was given by Hayes (1983) and Eigenbrode and Freeman (2006). They interpreted δ^13^C up to − 57 ‰ values in 2.8 to 2.6 Ga Hamersley sediments to methane cycling bacteria. Unfortunately, the δ^13^C range is ambiguous as some carbon fixation processes used by phototrophic bacteria, such as the Calvin cycle or hydroxypropionate pathways (Posth et al. 2013) or metamorphism (McKirdy and Powell 1974) produce overlapping δ^13^C ranges.

The stable iron isotope ratios (δ^56^Fe) in BIF of the Archean and early Paleoproterozoic may also be used to infer early photoferrotrophy. The δ^56^Fe values of BIFs vary widely between − 2.5 and 1.0 ‰ relative to values expected for lithogenic or hydrothermal sources (− 0.5 ‰ < δ^56^Fe < 0.3 ‰) (Czaja et al. 2013; Johnson et al. 2003, 2020). These BIF δ^56^Fe values differ from the homogenous values of modern marine sediments with δ^56^Fe values of 0.00 ± 0.05 ‰, and have been interpreted to reflect BIF minerals being both precipitated with equilibrium fractionation from an isotopically varying fluid, as well as indicating a microbial influence (Johnson et al. 2003, 2004; Johnson and Brian 2005; Steinhoefel et al. 2010). Planavsky et al. (2009, 2012) observed δ^56^Fe values of − 0.66 to + 0.82 ‰ in the 1.98 Ga old stromatolites of the Gunflint and Biwabik iron formations in the USA. Additionally, Johnson et al. (2003) observed highly negative δ^56^Fe values in BIF magnetite (up to − 0.57 ‰) from the Transvaal Supergroup, South Africa. Both analyses correlate with findings of experimental setups of ferric oxyhydroxides formed by photoferrotrophs, resulting in enrichment in the heavy iron isotope and δ^56^Fe values of 1.5 ± 0.2 ‰ (Croal et al. 2004). However, Fe(III) oxyhydroxides formed by chemical oxidation show comparable δ^56^Fe values, making it difficult to differentiate between biotic and abiotic processes (Bullen et al. 2001). Overall, it is considered that positively fractionated δ^56^Fe values reflect the partial oxidation of iron by either abiotic or biotic means. A recent examination of triple iron isotope systematics (Heard et al. 2020) also indicates that there was extensive iron oxidation in the Archean to Paleoproterozoic ocean. While there are uncertainties associated with such proxy approaches to discerning microbial activity, the wealth of independent evidence suggests that microorganisms were involved in BIF deposition, and build a strong basis for future work.

## The low concentrations of organic carbon in BIFs

One challenge to the apparent biogenicity of BIFs has been the low extent of organic carbon preservation, typically < 0.5 wt% (Gole and Klein 1981), and the extent of biomass-mineral coprecipitation has been a subject of considerable debate. If BIF deposition was indeed controlled by bacteria, biomass would be expected to have been sedimented in association with the iron minerals. Experiments by Posth et al. (2010) and Wu et al. (2014) show the close association of photoferrotrophic cells with Fe(III) minerals, while Swanner et al. (2015b) reported similar associations between cyanobacteria cells and Fe(III) minerals. However, Thompson et al. (2019) found that photoferrotroph biomass may be deposited separately from the formed Fe(III) minerals in the presence of silica, which could potentially explain the deposition of biomass-poor BIFs. Another possible explanation for the low organic carbon contents is that the biomass precipitated with minerals was then consumed by secondary microbiological processes (see discussion below) (Posth et al. 2013, 2014). Therefore, the lack of organic carbon in BIFs may be readily reconciled with the biological formation hypothesis.

## Diagenesis: secondary Fe(III) reduction

Based on the assumption that Fe(III) oxyhydroxides, such as ferrihydrite, were the sole primary mineral phase, one major question about the genesis of BIFs is: what processes were responsible for the formation of the reduced and mixed-valent minerals found in BIFs today? As stated earlier, BIFs are composed of secondary minerals that include magnetite, hematite, siderite, dolomite-ankerite, greenalite, stilpnomelane, and riebeckite (Bekker et al. 2010; Klein 2005; Pecoits et al. 2009), with an overall oxidation state of 2.4 (Klein and Beukes 1992). The mixed oxidation state of iron-bearing minerals, therefore, indicates the occurrence of secondary processes leading to the reduction of the primary ferrihydrite.

The mineralogy, petrology, and isotopic composition of BIFs provide evidence that microbial and metamorphic overprinting led to the observed BIF secondary mineral composition. BIFs with little metamorphic overprint provide a good opportunity to find evidence for microbial contribution to secondary mineral diagenesis. For example, microscopic, XRD, and Mössbauer spectroscopy analyses of magnetite in the 2.48 Ga Dales George BIF, Western Australia have shown the association of magnetite with Fe(III) acetate salt and nanocrystals of apatite, a signature often found in contemporary microbial magnetites (Li et al. 2011). Additional observations, such as highly negative δ^56^Fe values (− 0.57 ‰) (Johnson et al. 2003; Yamaguchi et al. 2005) in magnetite-rich BIFs (Heimann et al. 2010; Johnson et al. 2008; Reddy et al. 2016; Teixeira et al. 2017) point towards microbial Fe(III) reduction during mineral diagenesis. Walker (1984) was the first to propose that dissimilatory Fe(III) reducing bacteria (DIRB) were responsible for Fe(III) reduction in the primary BIF Fe(III) minerals, based on anomalously light δ^13^C values (on average a δ^13^C value of − 10 ‰) observed in the carbonate minerals in BIFs.

The secondary mineralogy also has been strongly influenced by high pressure and temperature, complicating the clear identification of microbial processes. For instance, Rasmussen and Muhling (2018) have proposed that the presence of magnetite in BIFs is a reflection of the thermal decomposition of siderite during metamorphism. In an effort to better constrain the potential role of microorganisms, or derivative organic matter, during BIF diagenesis, several laboratory studies have attempted to understand and distinguish biotic from abiotic processes that lead to alteration of the primary minerals.

## Experimental approaches to studying BIF mineral transformation

Some experimental studies aimed at deciphering secondary BIF mineral transformation have been concerned with dissimilatory Fe(III) reduction (DIR). Further studies have attempted to recreate the main mineral transformation pathways expected under metamorphic conditions by exposing primary Fe(III) minerals to high pressure and high temperature conditions that are representative of those unconsolidated BIF precursor sediment would have experienced during metamorphism.

Experimental studies by Beard et al. (1999) and Johnson and Brian (2005) showed that during microbial Fe(III) reduction, freshly formed dissolved Fe(II) was depleted in ^56^Fe by up to 3‰ compared to the Fe(III) oxyhydroxide source. Comparable δ^56^Fe values have been documented in magnetite-rich BIFs, suggesting that the magnetite might have been formed by microbial reduction (Johnson et al. 2003; Konhauser et al. 2005; Yamaguchi et al. 2005). Based on modelling, Konhauser et al. (2005) further suggested that, under ideal conditions, up to 70% of Fe(III) minerals could have been cycled back into the water column by DIR. This implies that DIRB could indeed have been a major player during the reduction of primary Fe(III) minerals in BIFs (Fig. 2:5).

Several laboratory studies have examined the characteristics of mineral precipitates formed by DIRB which may help identify microbially formed mineral phases. It has been shown that the identity and properties of the minerals formed by DIRB depend on (i) the amount of dissolved Fe(II) (Zachara et al. 2002), (ii) the concentration of nutrients and trace metals (Kukkadapu et al. 2004; Sergent et al. 2011), and (iii) the amount of organic matter (Amstaetter et al. 2012; Shimizu et al. 2013; ThomasArrigo et al. 2018; Zhou et al. 2018). An exemplary strain used to examine changes in ferrihydrite is the Fe(III)-reducing bacterium *Shewanella oneidensis* MR-1 (Han et al. 2020). For instance, Han et al. (2020) showed that magnetite can be formed by DIRB, albeit at a much slower rate than during the abiotic reaction of ferrihydrite and dissolved Fe(II). The sole exception to magnetite formation was abiotically reduced biotic ferrihydrite, in which case only goethite was formed. Recently, there has been the first approach to rebuild the microbial iron cycling during BIF deposition in the laboratory (own observation by Schad 2021). The marine photoferrotroph *Chlorobium sp.* N1 (Laufer et al. 2017) and a Fe(III)-reducing culture (Laufer et al. 2016) were cultivated in a media approximating Precambrian ocean composition (~ 4 mM Fe(II) and ~ 1.4 mM Si). During alternating oxidative and reductive cycles, a mixture of Fe(III) and Fe(II) minerals was formed. Overall, the range of secondary BIF minerals, such as magnetite and siderite, can be formed under various geochemical setups, supporting the hypothesis of microbially driven Fe(III) mineral reduction during the diagenesis of BIFs.

The secondary alteration of Fe(III) minerals by DIRB can only be assumed at temperatures lower than 120 °C, which is considered suitable for microbial activity (Kashefi and Lovley 2003). In addition, thermochemical mineral transformations during metamorphism have also been suggested to have influenced the secondary mineral (trans)formation during BIF formation in addition to microbial activity. Experiments reconstructing low-grade metamorphism by exposing Fe(III) minerals, admixed with glucose (as a proxy for microbial biomass) to 170 °C and 1.2 kbar for 14 days, formed mineral phases such as siderite, hematite, and magnetite (Köhler et al. 2013; Posth et al. 2013). When analyzed after prolonged storage under hydrated, oxidizing conditions, a similar experimental approach yielded hematite and lepidocrocite (Robbins et al. 2015). Instead of glucose, Halama et al. (2016) used more complex microbial biomass, and showed that under such conditions hematite and siderite but no magnetite were being formed. Additionally, it has been suggested that magnetite formed during thermal decomposition of siderite at 200–350 °C (Rasmussen and Muhling 2018). While this transformation may be applicable to highly metamorphosed BIFs that have undergone such conditions, it is unclear if magnetite found in less metamorphosed BIFs could have been formed via this process. Instead, the biogenicity of magnetite in BIFs is further supported by the results of Li et al. (2013) who demonstrated that biogenic magnetite can be preserved during low-grade metamorphism. This implies that minerals which were formed through microbial processes during sediment diagenesis would have been preserved during progressive low-grade metamorphism.

In summary, the presence of reduced or mixed-valence Fe-bearing minerals found in BIFs today may be explained by various formation pathways which are not mutually exclusive but likely rather represent a continuum of interacting processes. While highly metamorphosed BIFs have certainly undergone temperature and pressure-based mineral transformations, the magnetite in BIFs that experienced low-grade metamorphism is likely the result of early formation pathways (Li et al. 2011) and thus may show signs of biotic influence. Currently most hypotheses on BIF mineral diagenesis are built based on isolated reduction or oxidation processes. To better understand BIF deposition and post-depositional processes, it might thus be useful to link oxidation and reduction processes, in the form of Fe cycling experiments, potentially in combination with low-grade metamorphic incubations.

## Microbiological iron cycling: controlling factors and limitations

Several trace elements and nutrients such as nickel (Ni) and phosphorous (P) are capable of influencing microbial processes. Phosphorus, especially in the form of phosphate (PO_4_^3−^), is a direct limiting factor for microbial growth as it is an essential nutrient for most living creatures (Tyrrell 1999). Furthermore, P is often assumed to be the ultimate limiting nutrient with regards to primary productivity on geological time scales (Laakso and Schrag 2018; Tyrrell 1999). Nickel, on the other hand, is an essential trace metal cofactor for the enzymatic activity of methanogens (Zahnle et al. 2006; Zerkle 2005), and methanotrophs (Strous and Jetten 2004) and as such its availability might have influenced the redox evolution of the atmosphere–ocean system (Konhauser et al. 2009, 2015). Therefore, reconstructing the abundances and distribution of elements such as Ni and P through time, based in part of the BIF record, can provide insights into the evolution of their availability and the contemporary microbial community as well as the redox state of Earth’s ocean and atmosphere (see Robbins et al. 2016) for a review). Due to their high sorptive capacities, Fe(III) oxyhydroxides, such as ferrihydrite, are substantial sinks for trace elements and other ions in solution (Cornell and Schwertmann 2003; Dzombak and Morel 1990). Therefore, BIFs have the potential to provide critical insight into marine nutrient and trace metal concentrations in the ancient oceans (Bjerrum and Canfield 2002; Konhauser et al. 2009).

## Phosphorous

Phosphorous is an essential element in various biological processes and molecules. Besides its important role as a nutrient, phosphorous is part of the molecular structure of DNA and RNA, phospholipids, and cellular membranes. In general, high P concentrations in ancient rock formations often correlate to nutrient peaks in Earth’s history and, therefore, periods of biotic expansion (Jones et al. 2015; Planavsky et al. 2010). However, the exact concentration and role of P during BIF deposition, and the associated interplay with other elements, remains debated (Bjerrum and Canfield 2002; Jones et al. 2015; Konhauser et al. 2007; Planavsky et al. 2010). In terms of P concentration, BIFs are a good candidate for a proxy as they were deposited throughout much of the Archean and Paleoproterozoic, and P tends to readily adsorb on Fe(III) oxyhydroxides surfaces (Bjerrum and Canfield 2002; Kipp and Stüeken 2017). Based on the presumed ancient marine P concentrations, conclusions supporting either P limitation or P availability have been drawn with wide implications on marine conditions during this time period.

## BIFs as a proxy for ancient seawater phosphorous concentrations

**Fig. 3:**
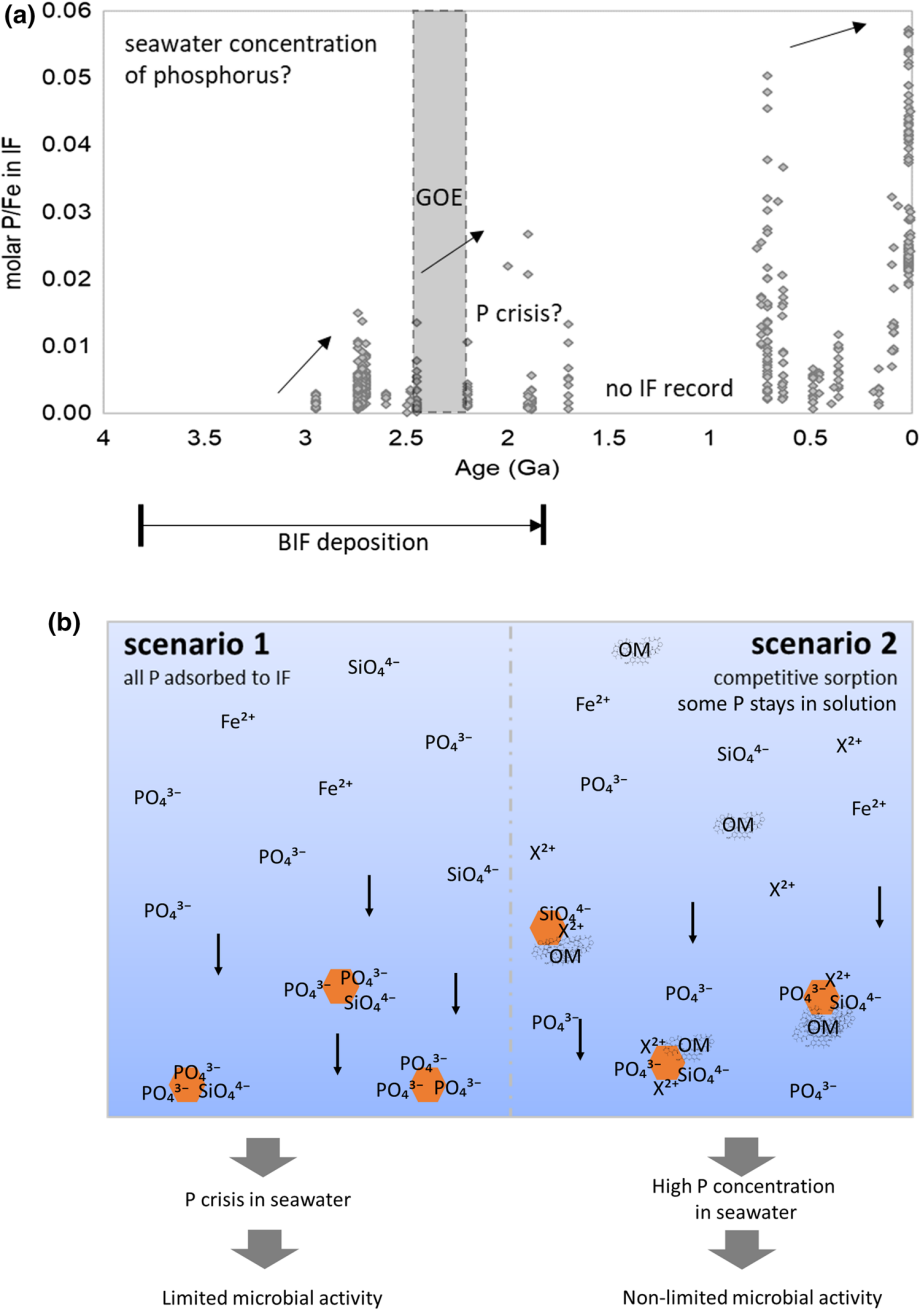
**a** Molar P/Fe concentrations in iron formations (IF) from 4 Ga to today, replotted using data from Robbins et al. (2016) and Planavsky et al. (2010); BIF deposition between 3.8 to 1.85 Ga, Great Oxidation Event (GOE) between 2.45 to 2.32 Ga; no IF record during the Mesoproterozoic gap between ~ 1.6 and ~ 0.7 Ga. Distal hydrothermal sediments are included to extend the record into the Phanerozoic. For sample filtering criteria see Planavsky et al. (2010). **(b)** Possible scenarios depicting the relationship between P contents in IFs and seawater P concentrations; scenario (1): all dissolved P (in the form of phosphate) adsorbs to Fe(III) minerals, therefore, the IF P/Fe ratio displays sea water concentration; scenario (2): a wide range of ions adsorb to ferrihydrite, some P stays in solution, therefore, sea water concentration of P is higher than indicated based the P/Fe ratio in the IF record as the surface of the ferrihydrite is passivated by the adsorption of other ions and less reactive to P; *X*^2+^  = divalent cations; orange hexagons = ferrihydrite

Most knowledge of P concentrations in ancient oceans is based on the P/Fe ratios in iron formations (IFs). IF contain not only BIFs but also granular iron formations that lack the typical banding (Robbins et al. 2016). Phosphorous to iron ratios through time are displayed in Fig. 3a (Planavsky et al. 2010; Robbins et al. 2016), and document fluctuations in the P/Fe ratios from 3 to 1.85 Ga and from 0.75 Ga to today. A characteristic lack of IF records results in the Mesoproterozoic gap (from ~ 1.6 to 0.7 Ga), but Fig. 3a includes distal hydrothermal sediments to extend the record into the Phanerozoic. The P/Fe ratios of IFs appear to show an overall increasing trend through time: P/Fe ratios appear to increase from ~ 0.005 at 3.0 Ga to ~ 0.015 at 1.85 Ga, and ultimately to modern values of between 0.05 and 0.06.

The interpretation of the P/Fe ratios in IFs is ambiguous. It is highly debated if lower P/Fe ratios prior to 1.85 Ga (Fig. 3a) reflect lower seawater P concentrations and if so, why the phosphorus concentrations during the time of BIF deposition were much lower than modern concentrations (~ 2.3 µM) (Bruland 1980; Bruland and Lohan 2003). Bjerrum and Canfield (2002) suggested that the low P/Fe ratios reflect low seawater P concentrations of between 0.15 to 0.6 µM and thus represent a P crisis (Fig. 3b scenario 1). This interpretation is based on the observation that P (in the form of phosphate) readily adsorbs to Fe(III) oxyhydroxides in the absence of competing ions. Such adsorption behavior of P on Fe(III) minerals has been observed near modern hydrothermal vents (Feely et al. 1990, 1998; Klein and Beukes 1992) and low P/Fe ratios would thus reflect low aqueous P concentrations.

Konhauser et al. (2007), however, challenged the hypothesis of a P crisis (Fig. 3b scenario 2), based on the high silica concentrations that would have characterized the ancient ocean (up to 2.2 mM) (Maliva et al. 2005; Siever 1992). For comparison, modern oceans have dissolved silica concentrations on the order of ~ 0.1 mM (Bruland and Lohan 2003). Experimental results presented by Konhauser et al. (2007) showed that Si effectively outcompetes P for sorption sites on Fe(III) oxyhydroxides, thus limiting P adsorption and resulting in the observed low P/Fe ratios in deposited iron minerals. In this case, the low P/Fe ratios would thus reflect much higher dissolved P concentrations but attenuated adsorption due to the influence of elevated marine silica concentrations (Konhauser et al. 2007). Planavsky et al. (2010) further inferred that the P concentrations at the time of BIF deposition might have been similar or even higher than modern concentrations. However, a subsequent study by Jones et al. (2015) showed that divalent cations such as Mg^2+^ and Ca^2+^ limit the influence that Si has on P adsorption. Based on their experimental results they suggested that early marine P concentrations would have ranged from 0.04 to 0.13 µM during IF deposition, which is 13 to 58 times lower than modern marine concentrations. Another factor to consider is the co-precipitation of organic matter with Fe(III) oxyhydroxides. Organic matter preferentially adsorbs to Fe(III) minerals compared to P (Li et al. 2011; Sundman et al. 2016; Yan et al. 2016), which, similar to Si, would potentially have resulted in higher dissolved P concentrations (see Fig. 3b). Collectively, these results suggest that although we have a good understanding of the individual factors influencing the initial adsorption of P to primary Fe(III) oxyhydroxides, the interpretation of the P contents in IFs remains elusive due to (i) lack of consensus on how multiple factors affect sorption, and (ii) a poor understanding of processes influencing post-deposition P mobility. The former also highlights the difficulty in drawing robust conclusions from the ancient rock record based on experimental, empirical models for trace element adsorption.

## Implications of oceanic phosphorus concentrations for early microbial life

The concentrations of P that limit microbial growth are still poorly constrained. Further, it is unclear how this limitation may have affected the potential role of cyanobacteria to BIF deposition. Depending on the exact interpretation of the BIF record (see above discussion on factors regarding adsorption), P may not necessarily have been a limiting factor. Jones et al. (2015) concluded based on P sorption experiments that phosphorus concentrations would have been as low as 0.04 to 0.13 µM. These concentrations are too low for cyanobacterial activity, and the authors suggested that such P-starved conditions were better suited to photoferrotrophs. Papineau et al. (2013) further stated that additional phosphorous from chemical oxidative weathering had to be washed into the ocean to support cyanobacterial activity. Conversely, as Konhauser et al. (2007) and Planavsky et al. (2010) interpreted concentrations similar to or higher than modern concentrations, P would not have been limiting for cyanobacterial growth. This interpretation is further supported by Rasmussen et al. (2021), who found high concentrations of the phosphate mineral apatite closely associated with greenalite in BIFs. Based on the assumption that greenalite, not Fe(III) oxyhydroxides, is the primary mineral in BIFs, this finding led to the conclusion that phosphate is also sourced from hydrothermal vent plumes and that the P concentration in seawater would have been considerably higher. An additional line of evidence in support of abundant P for microbial growth is the finding that DIRB may cause a release of 3–25% of adsorbed P via the reduction of Fe(III) minerals (Roden and Edmonds 1997). This potential release may have in turn benefited cyanobacteria or other P-limited microbes. Outstanding questions about P limitation may be addressed through experimental studies that target understanding phosphorus mobility during microbial Fe cycling experiments with co-cultures of cyanobacteria (or photoferrotrophs) and DIRB.

## Nickel

Nickel is a trace element that is critical to prokaryotic metalloenzymes and also part of several enzymes that participate in ureolysis, hydrogenotrophy, methanogenesis, and acetogenesis (Hausinger 1987). Particularly relevant to early Earth geobiology is methanogenesis; all known methane hydrogenases contain Ni. Further, the methyl coenzyme M reductase, found in all methanogenic bacteria, possesses a nickel-containing cofactor—F430. Thus, ancient marine concentrations of Ni may have directly controlled the abundance and distribution of methanogens. This, in turn, may have impacted atmospheric concentrations of oxygen (Konhauser et al. 2009, 2015). For example, a decrease in the available Ni would have inhibited the activity of methanogens, allowing for the spread of oxygen-producing cyanobacteria. In addition, Ni-containing hydrogenases are also known to be important for phototrophs (Hausinger 1987). Thus, the availability of Ni as a micronutrient in the ancient oceans has been interpreted as a control on methanogenic and phototrophic activity.

## BIFs as a proxy for ancient seawater nickel concentrations

Early efforts to determine nickel concentrations in the ancient oceans were based largely on geochemical modeling and indirect data such as the δ^34^S sulfide isotopic record (Saito et al. 2003). In both iron and sulfide-dominated ocean scenarios, seawater Ni concentrations have been suggested to be likely uniform through time (Saito et al. 2003). This interpretation was based on experimental evidence that Ni (i) is not a redox-sensitive element, and, therefore, would be relatively unaffected by oxic and anoxic transitions and (ii) that Ni and other trace metals react with sulfide, forming strong sulfide complexes that influence the bioavailability of metals and, therefore, the emergence of microorganisms. However, compilations of Ni data in BIF indicate nearly constant Ni/Fe ratios at about 5 × 10^–4^ from 4 Ga to 2.5 Ga, followed by a marked, unidirectional decline thereafter (Konhauser et al. 2009, 2015). The youngest BIFs (e.g., the Yerbal formation, Uruguay), from 0.5 Ga ago, show slightly increasing ratios up to 2 × 10^–4^. The decrease in Ni/Fe has been attributed to cooling of the mantle and the subsequent decrease in available Ni-rich rocks for weathering and transport (Konhauser et al. 2009, 2015; Liu et al. 2021).

The Ni/Fe ratios in BIFs have, in turn, been used to constrain dissolved Ni concentrations in the oceans through time. Such constraints are interpreted based on experimentally derived partitioning coefficients determined via laboratory-based experiments. The estimated dissolved Ni concentrations range from almost 40 to 400 nM in the Archean (dependent on dissolved silica concentrations) to values of 9 nM in the late Paleoproterozoic to Neoproterozoic (Konhauser et al. 2009). As the presence of silica suppresses Ni sorption and incorporation in a similar fashion to P, higher assumed silica concentrations result in higher estimates of dissolved Ni. In the case of 2.2 mM Si, the Ni concentration has been estimated to drop off sharply from 400 nm to < 200 nM around 2.5 Ga. This has significant implications for methanogens. Below 200 nM Ni, methanogenic activity has been shown to be severely limited (Kida et al. 2001; Schönheit et al. 1979). Further reports of Ni partitioning in biogenic ferrihydrite suggest that Ni concentrations may have been even higher in Archean oceans (Eickhoff et al. 2014), lending support to the hypothesis that methanogens were widespread. Evidence supporting the relevance of methanogenic and methanotrophic bacteria at the time of BIF deposition was also given by Hayes (1983) and Eigenbrode and Freeman (2006). They linked highly negative δ^13^C ratios between -57‰ to -37‰ in kerogen from the Hamersley province 2.8 to 2.6 Ga old sediments to methane cycling, which ultimately led to the deposition of the ^13^C depleted organic matter.

## Experimental work describing nickel partitioning and mobility

Several experimental studies have assessed the partitioning and subsequent diagenetic mobility of nickel adsorbed to ferrihydrite in order to quantify Ni retention and mobility. Konhauser et al. (2009) demonstrated that the presence of silica decreases the amount of adsorbed Ni to ferrihydrite. This is supported by (Eickhoff et al. 2014), who examined the sorption of Ni during co-precipitation of Ni in presence of dissolved silica with ferrihydrite by freshwater photoferrotrophs (*Rhodobacter ferrooxidans* strain SW2) and marine photoferrotrophs (*Rhodovulum iodosum*). The authors found that the adsorption of Ni to ferrihydrite was lower to biogenic minerals in comparison to abiotic minerals. This lead to the hypothesis that Ni concentrations in seawater at the time of BIF formation might have been higher than previously interpreted (up to 400 nM (Konhauser et al. 2009)).

Robbins et al. (2015) found evidence of similar sorption behavior of Ni to biogenic and abiogenic ferrihydrite, and further examined the mobility of Ni during subsequent temperature–pressure incubations designed to simulate diagenetic to low-grade metamorphism mineral transformations. Abiotic ferrihydrite and biogenic ferrihydrite, with high concentrations of adsorbed Ni, were incubated at 170 °C and 1.2 kbar in the presence or absence of glucose. Following incubation, 93% of Ni was retained on biogenic ferrihydrite and 91% on abiogenic ferrihydrite. In summary, iron cycling is a possible pathway to mobilize a small percentage of Ni initially adsorbed to BIF. However, to better quantify how much Ni may be released or taken up during formation and diagenesis, further studies should include experiments specifically designed to investigate the cycling of Ni between the dissolved pool and iron mineral phase under conditions representative of ancient oceans, as well as the mobility during higher degrees of metamorphism.

## Conclusions and perspectives

Our knowledge on BIF genesis is based on a combination of theoretical and geochemical models, analysis of the ancient rock record, and laboratory simulations of early Earth conditions. Although the lack of direct micro fossil evidence makes it difficult to unambiguously identify a microbial role in BIF deposition, several independent lines of argument point towards the biogenicity of BIFs. Precambrian ocean geochemistry, defined by its anoxic, iron and silica-rich environment, has been shown to be hospitable for several microbial metabolisms, including cyanobacteria, photoferrotrophs, and DIRB based on experimental studies. These microorganisms may have been the key players in Precambrian iron cycling in marine systems, and ultimately led to the mineralogy we find in BIFs today. Accordingly, abiotic deposition mechanisms, such as direct Fe(II)-silica mineral precipitation (instead of Fe(III) minerals) and photooxidation of Fe(II), may have played a much smaller role than recently suggested. The precipitation of BIFs by the biotic or abiotic oxidation of dissolved Fe(II) would further support their continued use as proxies for reconstructing ancient nutrient and trace metal levels, as has been the case for phosphorus and nickel. Phosphorus scarcity (leading to the inhibition of microbial activity), or nickel abundance (stimulating counteracting methanogens, producing methane, which acts as a sink for oxygen), can be at least partly ruled out by recent experimental work. Previous research on elucidating the role of microorganisms in BIFs has focused mostly on individual Fe(II)-oxidizing or Fe(III)-reducing processes. The combination of these processes, however, is critically important. Future experiments aimed at recreating Fe cycling during BIF deposition with consortia of Fe(II)-oxidizing and Fe(III)-reducing bacteria, coupled with further investigations of nutrient and trace metal mobility in presence of different iron minerals, are necessary to discern processes occurring during the deposition and diagenesis of BIFs and better interpret early Earth conditions.
